# Transcriptomic profile of the zoonotic parasite *Anisakis pegreffii* upon *in vitro* exposure to human dendritic cells

**DOI:** 10.3389/fcimb.2025.1646537

**Published:** 2025-09-15

**Authors:** Marialetizia Palomba, Aurelia Rughetti, Tiziana Castrignanò, Chiara Napoletano, Xavier Roca-Geronès, Valentina Pinna, Franco Liberati, Daniele Canestrelli, Simonetta Mattiucci

**Affiliations:** ^1^ Department of Ecological and Biological Sciences, “Tuscia University”, Viterbo, Italy; ^2^ Department of Experimental Medicine, Sapienza University of Rome, Rome, Italy; ^3^ Department of Biology, Health and Environment, Section of Parasitology, Faculty of Pharmacy and Food Sciences, University of Barcelona, Barcelona, Spain; ^4^ Department of Public Health and Infectious Diseases, University Hospital “Policlinico Umberto I”, Sapienza-University of Rome, Rome, Italy

**Keywords:** human anisakiasis, *Anisakis pegreffii*, human dendritic cells, RNA-seq, gene expression, oxidative stress, energy metabolism

## Abstract

*Anisakis pegreffii* is a zoonotic marine nematode whose life-cycle involves marine organisms–small crustaceans, fish and squids as intermediate/paratenic hosts, and marine mammals, mainly cetaceans–as definitive ones. When its third-stage larvae (L3) are accidentally ingested by humans with the consumption of raw or undercooked parasitized fish and/or squids, the parasite fails to complete its life cycle, leading to human anisakiasis. Despite increasing interest in understanding the molecular basis of pathogenic effects in human anisakiasis, the transcriptomic response of *A. pegreffii* L3 to interaction with human immune cells, remains poorly understood. Thus, in this study, the transcriptomic profile of *A. pegreffii* L3 larvae under *in vitro* exposure to human dendritic cells (DCs) was performed for the first time. A total of 3914 differentially expressed genes (DEGs) were identified in *A. pegreffii* L3 after exposure to immature DCs (iDCs), by RNA-seq, allowing to detect 1868 upregulated and 2046 downregulated transcripts. Upregulated genes were significantly enriched in pathways related to energy metabolism, oxidative stress response and structural maintenance, suggesting active metabolic and structural adaptation to immune-induced stress. Conversely, genes involved in cytoskeletal organization and intracellular trafficking were downregulated, potentially reflecting the parasite’s developmental arrest in an unsuitable host such as humans. These findings provide novel insights into the molecular response pathways activated by this zoonotic parasite during the early stages of interaction with the human immune system.

## Introduction

*Anisakis pegreffii* is a zoonotic nematode within the *A. simplex* (s. l.) complex, with an indirect life cycle involving cetaceans as definitive hosts, and small crustaceans, teleost fish, and cephalopods as intermediate/paratenic hosts (Mattiucci et al., 2018; 2022). Humans become accidental hosts by consuming raw or undercooked fish or squids infected with live third stage larvae (L3) of *A. pegreffii* (Mattiucci et al., 2018; Guardone et al., 2018). In the human, however, the parasite is unable to complete its development (Trumbić et al., 2021) but it can trigger inflammation leading to acute gastrointestinal symptoms and, if not promptly removed, marked eosinophilic infiltration, with abscess and granuloma formation (Nieuwenhuizen and Lopata, 2013; Mattiucci et al., 2013; Trumbić et al., 2021). Exposure to *Anisakis* antigens may also elicit IgE hypersensitization, provoking allergic reactions (Nieuwenhuizen and Lopata, 2013; Mattiucci et al., 2013; Brusca et al., 2023; EFSA, 2024). The pathogenicity of *A. pegreffii* is likely driven by a combination of factors, involving both mechanical invasive capacity and the release of excretory/secretory products (ESPs) (Mehrdana et al., 2017)—some of which are transported *via* extracellular vesicles (EVs) (Cavallero et al., 2022; Palomba et al., 2023) and miRNA (Cavallero et al., 2022; Bellini et al., 2025) —as well as complex interactions with host immune cells (Maizels and McSorley, 2016). Recent transcriptomic studies in a rodent model have shown that *A. pegreffii* L3 larvae upregulate genes associated with ribosomal function and oxidative phosphorylation, potentially indicating a failed attempt to molt (Trumbić et al., 2021). In contrast, in natural heterothermic fish hosts, autophagy-related pathways are activated, suggesting a dormancy-like state, highlighting the parasite’s ability to modulate its gene expression in response to diverse host environments (Trumbić et al., 2021).

However, very little is known about how *A. pegreffii* responds to specific components of the human immune system. Dendritic cells (DCs), as antigen-presenting cells (APCs), play a pivotal role in initiating cellular immunity (Maizels and McSorley, 2016; Lundie et al., 2016; Peng et al., 2022). Generally speaking, upon exposure to helminth-derived products, DCs undergo phenotypic changes that promote the triggering of a Th2-polarized immune response, contributing to immune tolerance; this response is frequently associated with elevated IgE levels, eosinophilia, and mast cell activation (Sher et al., 2003). Previous *in vitro* studies have shown that co-cultures of DCs with *A. pegreffii* L3 can impair DC function by reducing their maturation and immunogenic potential (Napoletano et al., 2018). Similarly, antigens from *A. simplex* (s. s.) larvae have been shown to suppress the expression of costimulatory molecules on DCs while promoting Treg expansion and modulating IL - 10 and IFN-γ production in a host-genotype-dependent manner (Zamora et al., 2019; 2021). However, the reciprocal interaction between the parasite and DCs remains poorly understood—both in terms of how DCs are modulated by the parasite and how the parasite’s gene expression responds to this key immune cell type during their interaction.

Therefore, this study aims to characterize the transcriptomic response of *A. pegreffii* L3 larvae during *in vitro* exposure to human DCs, in order to better understand early parasites’ molecular interactions with components of the human immune system.

## Materials and methods

### *Anisakis* L3 sampling

*Anisakis* L3 larvae were carefully removed from the body cavity of European hake (*Merluccius merluccius*) specimens, caught approximately 12h before, from the Adriatic Sea (off San Benedetto del Tronto coast)–a fishing area with a known high prevalence of *Anisakis* infection (Cipriani et al., 2018). To control for variability and minimize possible host-related effects, larvae were collected from three different *M. merluccius* individuals. After their removal, L3 were checked for their integrity and viability under a dissecting microscope. A viable larva in this context was considered physically intact and motile, as measured in terms of its ability to move spontaneously or by stimulation with tweezers and a needle (EFSA, 2024). Alive and not disrupted larvae were washed in a sterile 1X phosphate-buffered saline solution (PBS, Sigma, St Louis, MO) several times and then treated for 1 min with 4% acetic acid (Carlo Erba, Cornaredo, Italy) to inhibit bacterial contamination.

### Molecular identification of *Anisakis* spp.

*Anisakis* L3 were identified by using a multilocus molecular approach. The mitochondrial cytochrome c oxidase 2 (mtDNA *cox*2) gene locus was amplified using the primers 211F (forward; 5′-TTTTCTAGTTATATAGATTGRTTYAT-3′) and 210R (reverse; 5′- CACCAACTCTTAAAATTATC - 3′) (Valentini et al., 2006; Mattiucci et al., 2014). The successful PCR products were purified, and Sanger sequenced through an Automated Capillary Electrophoresis Sequencer 3730 DNA Analyzer (Applied Biosystems), using the BigDye^®^ Terminator v3.1 Cycle Sequencing Kit (Life Technologies). Additionally, a direct genotyping determination of the nuclear metallopeptidase 10 gene locus (*nas*10 nDNA) was performed by the amplification-refractory mutations system (ARMS) PCR assay at *nas*10 nDNA by the combined use of OUT-F1 (forward; 5’- TATGGCAAATATTATTATCGTA - 3’), OUT-R1 (reverse; 5’-TATTTCCGACAGCAAACAA-3’), INN-F1 (forward; 5’-GCATTGTACACTTCGTATATT-3’), INN-R1 (reverse; 5’-ATTTCTYCAGCAATCGTAAG-3’), following the procedures reported in Palomba et al., 2020. PCR products were separated by electrophoresis using agarose gel (1.5%) stained with GelRed. The distinct banding patterns were detected using ultraviolet transillumination.

### Exposure of *A. pegreffii* L3 to iDCs

For exposure of *A. pegreffii* L3 to DCs, immature DCs (iDCs) were generated by immunoselected CD14+ monocytes cells obtained from healthy donors, in presence of GM-CSF and IL - 4 as previously described (Dionisi et al., 2018; Napoletano et al., 2007). Each iDCs culture underwent quality control assessment before being used with *A. pegreffii* (File S1, **Supplementary Figure S1**). iDCs-L3 larvae co-cultures were established, as previously described (Napoletano et al., 2018). iDCs and L3 larvae were co-cultured in transwell plate (C24 Transwell plate, Corning Costar): L3 larvae were placed in the upper chambers (7 larvae/mL), while iDCs were seeded in the lower chamber (6x10^5^ cells/mL). In detail, a total of 63 L3 larvae were used for the co-culture condition, distributed across nine upper chambers, with 7 larvae per each chamber. Each upper chamber was paired with a corresponding lower chamber containing iDCs at a density of 6×10^5^ cells/mL. To account for biological variability, every three chambers shared iDCs from the same healthy donor, resulting in three experimental groups: Chambers 1-3: Paired with iDCs from Donor 1; Chambers 4-6: Paired with iDCs from Donor 2; Chambers 7-9: Paired with iDCs from Donor 3. As a control, an additional set of nine chambers was prepared under the same conditions but without iDCs, maintaining 7 L3 larvae per chamber. After 24 h, the surviving *Anisakis* L3 were collected, washed in PBS (twice), and stored in RNA later at -80°C for DNA and RNA extraction.

### Ethics statement

All research was conducted in accordance with relevant guidelines and regulations. The study protocol was approved by the Ethics Committee of University Hospital “Policlinico Umberto I” ‘Sapienza’ University of Rome (Protocol nr. 4212). Peripheral blood mononuclear cells (PBMCs) were isolated by Ficoll-Hypaque gradient (1.077 g/mL; Pharmacia LKB, Uppsala, Sweden) from buffy coats of healthy donors obtained from the Transfusion Center, Policlinico Umberto I. Formal written informed consent was obtained from all the healthy blood donors, prior to blood sample collection. Research involving human participants was conducted in compliance with the Declaration of Helsinki.

### DNA/RNA extraction

Both DNA and RNA were extracted from each *Anisakis* larva using TRIzol reagent (Invitrogen, Carlsbad, CA, USA), as previously described (Palomba et al., 2019). Specifically, one larva was collected from each chamber for extraction. This resulted in 9 larvae from the co-culture condition with iDCs (3 larvae for each donor, from different chambers), 9 larvae from the control condition without iDCs (1 larva for each chamber). DNA obtained from each larva was used for species-level identification, while RNA was treated with DNase (DNase I, Invitrogen) to remove any genomic DNA contamination. Then, treated RNA from three larvae (each obtained from a separate chamber with iDCs from different donators) was pooled together. Pooling of larvae was performed to reduce the impact of individual-level transcriptional noise and to obtain a representative average expression profile of *A. pegreffii* larvae under each experimental condition. This process was repeated three times (3L3/pool, 3 pools in total) to provide triplicate samples for RNA sequencing. RNA concentration, purity and integrity were verified and measured on agarose gel (2%) and by a Bioanalyzer 2100 (Agilent Technologies, Waldbronn, Germany).

### Library preparation and RNA sequencing

Following the manufacturer’s protocol, the cDNA library was prepared using a TruSeq Stranded mRNA kit (Illumina, San Diego, USA). In brief, polyadenylated (PolyA+) RNA was purified from 10 μg of total RNA of *A. pegreffii* L3 using Sera-Mag oligo (dT) beads, fragmented to a length of 100 - 500 nucleotides and reverse transcribed to cDNA using random hexamers. The size-fractionated cDNA was end-repaired and adaptor-ligated according to the manufacturer’s protocol (Illumina). Ligated products of 200 bp were excised from agarose gels and PCR amplified. Products were cleaned using a MinElute PCR purification kit (Qiagen Hilden, Germany) and single-end sequenced on an Illumina HiSeq 2000, according to the manufacturer’s protocol.

### Transcript quantification

The raw sequences obtained were processed using Salmon software (v. 1.5.1) (Patro et al., 2017) for mapping against the *A. pegreffii de novo* reference transcriptome, available online at figshare (https://figshare.com/articles/online_resource/AP_-_Unigenes/18301772) (Palomba et al., 2022). This process was conducted in two distinct phases: 1) the index command of Salmon was used to generate an index of the reference transcriptome; 2) the quant command was used to quantify the abundance of transcripts in the analyzed samples. Both steps were carried out using the software’s default parameters. The transcript quantification phase was performed on a High-Performance Computing Cluster (Castrignanò et al., 2020; Flati et al., 2020). We applied the TransDecoder tool to the Corset-assembled transcriptome, to predict Open Reading Frames, generating peptide (AnisakisPegr:Longest_ORFs_PEP.fasta) and coding sequence (AnisakisPegr:Longest_ORFs_CDS.fasta) files.

### Differential gene expression analysis

Differentially expressed genes (DEGs) were identified using IGUANER (Pinna et al., 2024), a software based on DESeq2algorithm (Love et al., 2014). DEGs were defined based on adjusted p-values (padj< 0.05) and log2 fold change thresholds. For the functional annotation of DEGs identified with IGUANER, we adopted two complementary approaches. Initially, we used DIAMOND software (v. 2.0.11) (Buchfink et al., 2014) to conduct sequence comparisons against the Nr, SwissProt, and TrEMBL databases. This step allowed us to obtain highly accurate and relevant homology-based annotations for each analyzed sequence. As second approach, we used Eggnog Mapper (v. 2) (Cantalapiedra et al., 2021) to perform comparisons of sequences with a broad range of homologous groups catalogued in the EggNOG database. This process enabled the acquisition of detailed functional annotations on the basis of the information contained in Gene Ontology (GO) database. A heatmap was generated to visualize the expression patterns of genes based on GO enrichment analysis. Briefly, DEGs from the GO enrichment analysis were filtered to remove duplicates, categorized into up- and down-regulated genes, and merged into a single dataset. The expression data were extracted from the annotated transcriptome file, filtered for significant DEGs (padj< 0.05, |log2FC| > 1), and visualized after eliminating redundant entries.

The complete bioinformatics pipeline is reported in **Supplementary Figure S2**. All results obtained from the bioinformatics analyses were deposited on Figshare (**Table 1**). From the complete list of DEGs (padj< 0.05, |log2FC| > 1), genes were selected based on their upregulation and relevance to detoxificant/antioxidant processes as well as chaperone and allergenic properties. This selection was performed manually, guided by existing literature and known gene functions.

**Table 1:** Overview of produced data files and their access on Figshare.

Label	Name of data	Data repository (URL)
Data file 1	*Anisakis pegreffii* trascriptome ORF (FASTA)	https://doi.org/10.6084/m9.figshare.27894762
Data file 2	*A. pegreffii* DEGs (FASTA)	https://doi.org/10.6084/m9.figshare.27894789
Data file 3	*A. pegreffii* EggNog result on trascritpome ORFs (*Ansakis pegreffii* EggNog result on trascritpome ORFs.tsv and Anisakis_pegreffi_orfs_Emmapper.xlsx)	https://doi.org/10.6084/m9.figshare.27896766
Data file 4	*A. pegreffii* DESeq2 results	https://doi.org/10.6084/m9.figshare.27894798
Data file 5	*A. pegreffii* Homology Annotation	https://doi.org/10.6084/m9.figshare.27891237
Data file 6	*A. pegreffii* transcriptome output (pep,cds)	https://doi.org/10.6084/m9.figshare.28113566
Image 1	*A. pegreffii* Functional Annotation (images)	https://doi.org/10.6084/m9.figshare.27894819

### Quantitative real-time PCR validation

To validate the results of RNA-seq, six differentially expressed genes were randomly selected for quantitative real-time PCR (RT-qPCR). The RNA samples used for the RT-qPCR assay were the same as those used for RNA-seq. The cDNA was synthesized for the RT-qPCR using the high-capacity cDNA Reverse Transcription Kit (Thermo Fisher Scientific, Wilmington, DE, USA) following the manufacturer’s instructions. The RT-qPCR was performed using SYBR Green PCR Master Mix, according to the manufacturer’s instructions. Amplifications were conducted for p-glycoprotein 2, tetraspanin, carboxypeptidase, ras-related protein Rab, galectin, aspartic protease 6, and elongation factor (EF) as reference gene. Specific primer pairs were designed (**Supplementary Table S1**), and standard curves were generated. All reactions were performed in triplicate in the StepOnePlus Real-Time PCR Detection System (Applied Biosystems) and relative quantification was carried out with the ΔΔCT method (Livak and Schmittgen, 2001) using the abundance of EF mRNA as endogenous housekeeping control. The relative transcription levels as obtained by RT-qPCR analyses were compared with abundance levels detected by RNA-seq. Values from replicate experiments were averaged. Finally, to obtain values suitable for statistical comparisons, the fold change (FC) value was calculated. These values (plotted after conversion in log2 numbers) were used to evaluate the correlation between RNA-seq and RT-qPCR methods, applying statistical evaluation using the Pearson test (in Prism GraphPad software).

## Results

### Molecular identification of *A. pegreffii* L3

A multilocus genotyping approach, combining mitochondrial and nuclear markers, was applied to ensure species identification (Mattiucci et al., 2018, 2025). BLAST analysis of the 21 sequences obtained from the *Anisakis* L3 at the mtDNA *cox*2 gene locus (~600 bp) retrieved a percentage of identity of 99 - 100% with the sequences of *A. pegreffii* previously deposited (accession numbers, KY565564-KY565562). Additionally, ARMS-PCR analysis targeting the *nas10* locus generated a species-specific 117 bp band, diagnostic of the C-allele, further confirming the identification of *A. pegreffii* (Palomba et al., 2020).

### Sequencing statistics

The quality of extracted RNA from L3 reached the standard of sequencing with RIN values >7, and the concentration of each sample was higher than 50 ng/μL. The total number of raw reads obtained by sequencing was 154047207, while the total number of clean reads after filtration was 153572782 (**Table 2**). The total number of clean read bases was 9580666822. Q30 was greater than 97.13%, and GC content was 43.02-46.81% (**Table 2**).

**Table 2:** Summary of data output quality of various libraries.

Sample name	Treatment	# raw reads	# trimmed reads	# nucleotides in trimmed reads	Q30 (%) after trimming	GC (%) after trimming	# raw reads mapped on transcriptome (%)
A2	*A. pegreffii* without DCs	27880321	27793506	1733338967	97.13	46.35	27569543 (98.89%)
A3	*A. pegreffii* without DCs	22795434	22723719	1418033788	97.19	46.65	22557881 (98.96%)
B2	*A. pegreffii* without DCs	24071703	23991979	1496859029	97.15	46.81	23817688 (98.95%)
C2	*A. pegreffii* with DCs	27798561	27716076	1729116693	97.25	43.72	27528318 (99.02%)
C3	*A. pegreffii* with DCs	24772741	24698236	1540686228	97.26	43.41	24449461 (98.70%)
D2	*A. pegreffii* with DCs	26728447	26649266	1662632117	97.27	44.00	26464488 (99.01%)

The raw data of all samples reported in this study were deposited in the NCBI (National Center for Biotechnology Information) under the accession number PRJNA752284.

### Differential gene expression in *A. pegreffii* following iDC exposure

**Figure 1:**
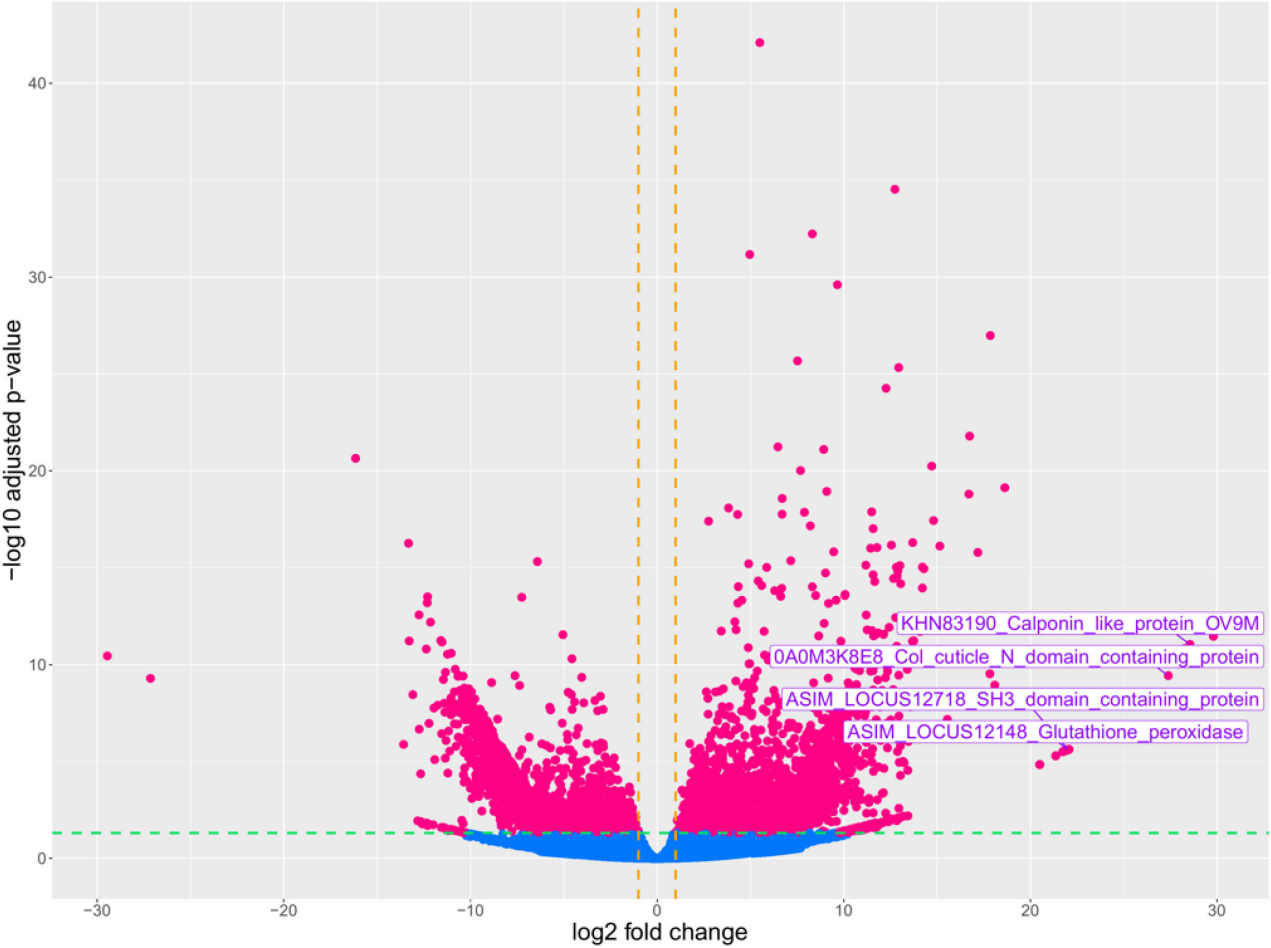
Volcano plot of DEGs between the treatment group (*A. pegreffii* L3 in the presence of iDCs) and control group (*A. pegreffii* in the absence of iDCs). The plot represents gene expression levels, with log2 Fold Change displayed on the x-axis and log10 (p.adjust) on the y-axis. The vertical and horizontal dashed lines indicate the fold-change cut-off = ± 1 and the p-value = 0.05, respectively. Pink dots indicate up and down-regulated genes, and light blue dots represent genes without significant changes in expression.

A total of 3914 differentially expressed genes (DEGs) with|log2FC| > 1 and p.adj< 0.05 were detected in the *A. pegreffii* L3 larvae treatment group (L3 co-cultured with iDCs) compared to the control group (L3 alone) (**Supplementary Table S2**). Results from DEG counts are reported in **Table 3**. Among DEGs, 1868 genes were up-regulated in L3 plus iDC compared to L3 alone, while 2046 genes were down-regulated (**Figure 1**). The most significantly modulated genes, characterized by the highest log2 fold change values, are highlighted in the plot (**Figure 1**). Notably, these included: glutathione peroxidase, SH3 domain-containing protein, calponin-like protein, and collagen cuticle N domain-containing protein, which exhibit strong differential expression, i.e., 22.00, 22.04, 28.55, 27.39 log2 fold change, respectively.

**Table 3:** Overview of differentially expressed gene counts across log2FC thresholds, padj ≤ 0.05.

log2FC Threshold	Total DEGs	Up-regulated DEGs	Up-regulated DEGs annotated in NR	Up-regulated DEGs annotated in TrEMBL/Swiss-Prot	Down-regulated DEGs	Down-regulated DEGs annotated in NR	Down-regulated DEGs annotated in TrEMBL/Swiss-Prot
|log2FC| ≥ 1	3914	1868	927	941	2046	1033	1013
|log2FC| ≥ 2	3478	1637	811	826	1841	919	922
|log2FC| ≥ 5	2389	1001	469	532	1388	699	689
|log2FC| ≥ 10	471	360	162	198	111	55	56

*NR: NCBI’s non-redundant (NR) database

### Functional enrichment and biological relevance of DEGs

**Figure 2:**
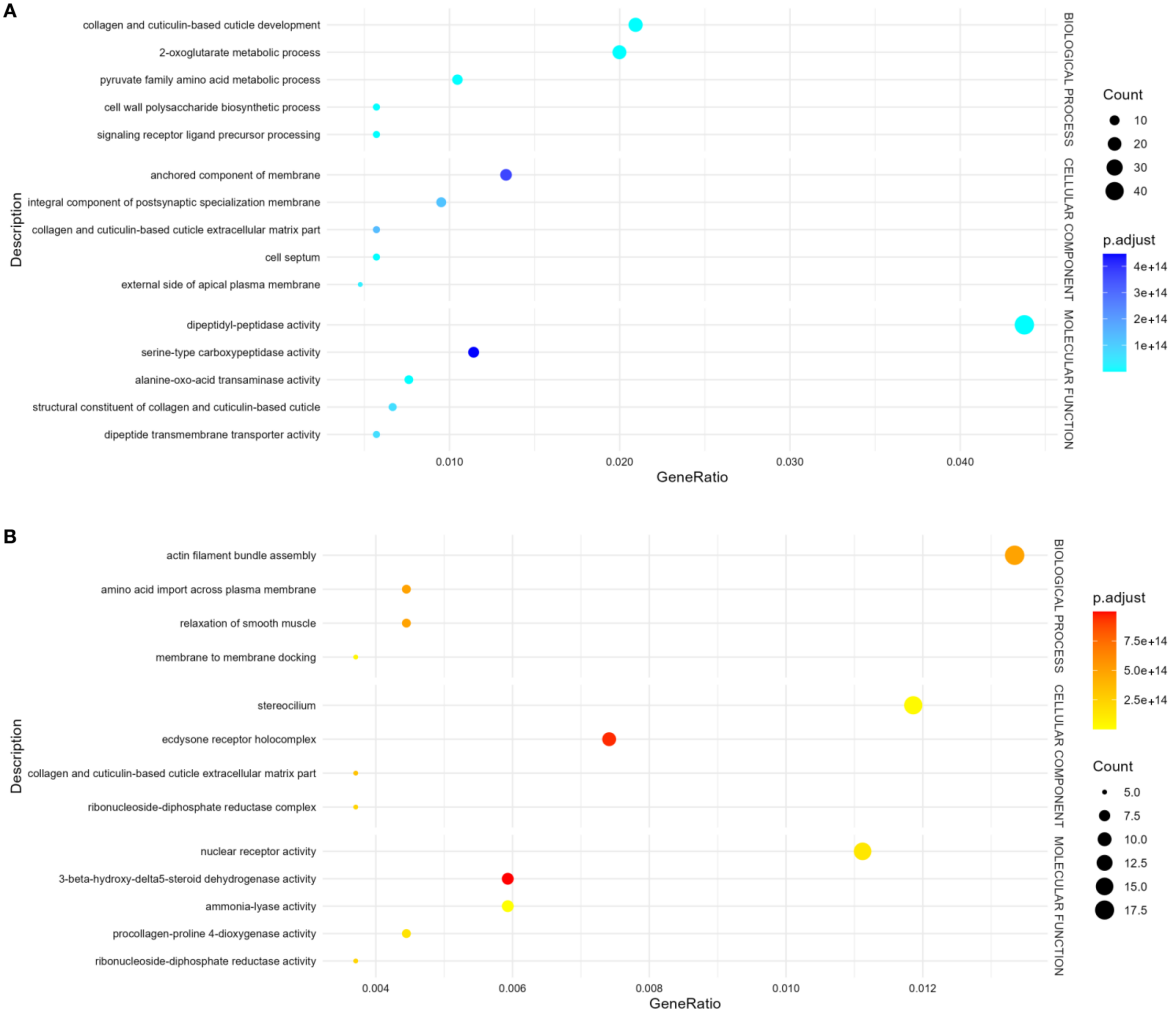
Bubble plot of GO terms enrichment analysis of DEGs. “Count” indicates the number of DEGs enriched in the pathway; “GeneRatio” indicates the ratio of enriched DEGs to background genes; “p.adj”, shown by color, indicates a significant level of enrichment results. **(A)** Up-regulated genes, **(B)** Down-regulated genes.

**Figure 3:**
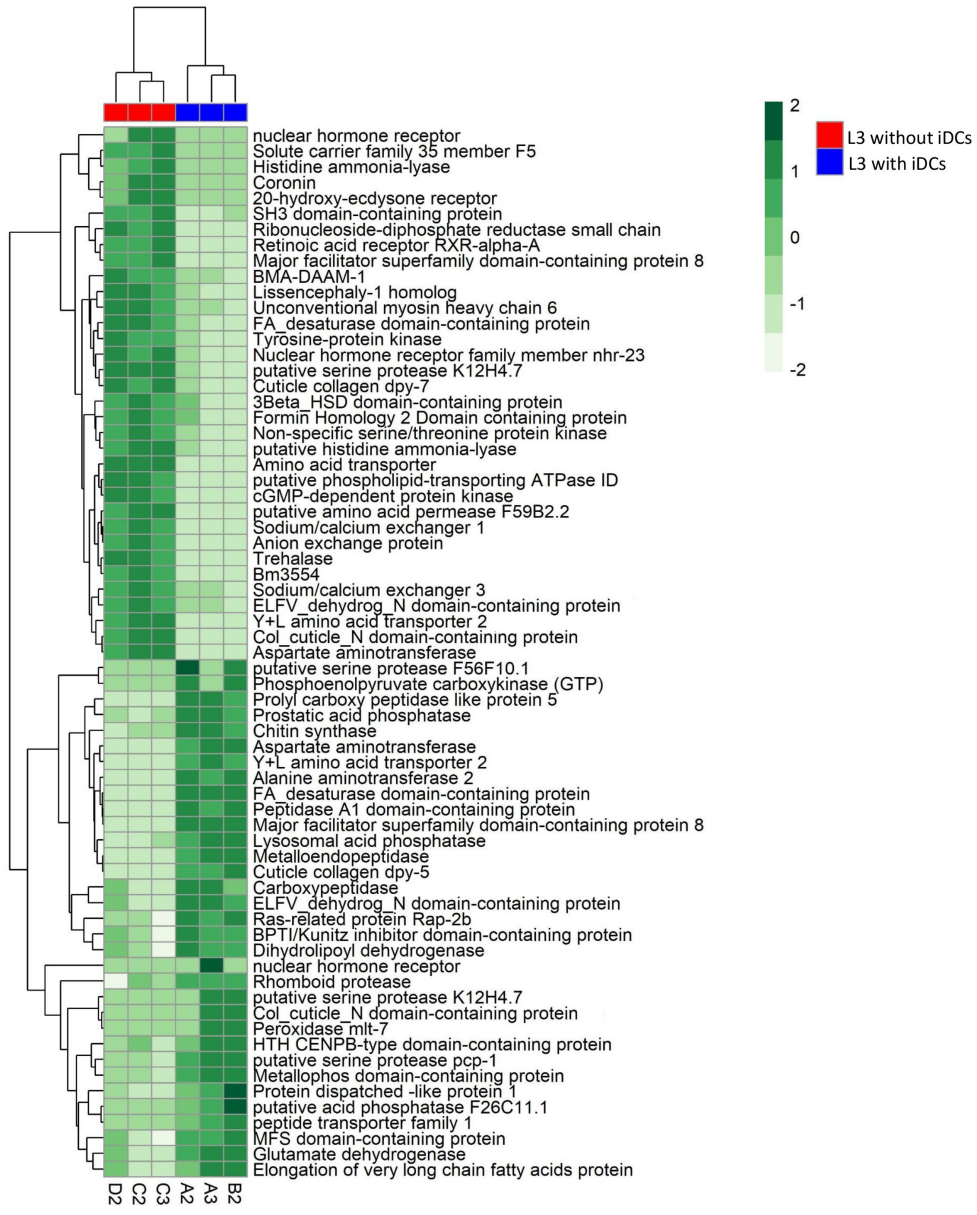
Heat map showing the log_2_ fold change of genes enriched in GO ontology categories in *A. pegreffii* L3.

**Figure 4:**
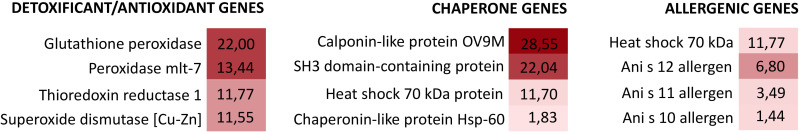
Heat map showing the log_2_ fold change of upregulated genes in *A. pegreffii* L3 within selected specific categories.

To better understand the functional implications of the transcriptional changes, we conducted GO enrichment analysis on up- and down-regulated DEGs. GO enrichment results revealed significantly enriched categories (p.adj< 0.05) (**Figure 2**, **Supplementary Table S3**). Up-regulated genes were mostly involved in collagen and cuticulin-based cuticle development (BP), dipeptidyl-peptidase activity (MF), and anchored component of membrane (CC) (**Figure 2A**, **Supplementary Table S3**). Conversely, down-regulated genes were enriched in actin filament bundle organization (BP), nuclear receptor activity in (MF), and stereocilium bundle (CC) (**Figure 2B**, **Supplementary Table S3**). The heatmap (**Figure 3**) visualizes the expression patterns of the GO-annotated genes identified in the enrichment analysis. In addition, we specifically examined the list of DEGs (**Supplementary Table S2**) to identify genes of relevance. Further analysis identified the upregulation of genes associated with detoxification and antioxidant processes (e.g., glutathione peroxidase, thioredoxin reductase 1, peroxidase mlt-7, and superoxide dismutase), allergenic responses (e.g., heat shock protein 70, anis12, anis11, and anis10), and chaperone-related functions (e.g., calponin, SH3 domain-containing protein, HSP70, and HSP60) (**Figure 4**). Comparison of the DEGs identified with those previously reported in the extracellular vesicles (EVs) of *A. pegreffii* (Palomba et al., 2022) revealed 21 overlapping genes (**Supplementary Table S4**).

### Correlation between RNA-seq and RT-qPCR results

The abundance of transcripts emerging from transcriptomic analyses was confirmed by RT-qPCR by examining six transcripts differentially modulated in *A. pegreffii* L3 in the presence/absence of iDCs i.e. p-glycoprotein 2, tetraspanin, carboxypeptidase, ras-related protein Rab, galectin, and aspartic protease 6. The validation analysis was performed using triplicate RNA samples. Correlation analysis indicated significant linear relationships (R2 = 0.70; p< 0.03) between RNA-seq and RT-qPCR results (**Table 4**, **Supplementary Figure S3**).

**Table 4:** Gene expression levels obtained by RNAseq and RT-qPCR.

Genes	Description	RNAseq (log_2_ Fold Change)	RT-qPCR (ΔΔCt method)
*phly*	p-glycoprotein 2	-9.23	-1
*car*	Carboxypeptidase	-4.37	-4.66
*gal*	Galectin	5.95	4.56
*ap6*	Aspartic protease 6	9.96	3
*tr*	Tetraspanin	-4.88	-3.25
*ras*	Ras-related protein Rab	10.66	4

## Discussion

Throughout their life cycle, helminth parasites are exposed to diverse microhabitat conditions within their hosts, including fluctuations in temperature, pH, nutrient availability, and host immune pressures. Among these, the interaction with the host immune system is particularly critical for parasite survival, especially in accidental hosts, such as humans, which represent an evolutionary dead-end and an unsuitable ecological niche for *A. pegreffii* (Trumbić et al., 2021), placing the parasite at a disadvantageous position. In this case, the host immune system rapidly recognizes the parasite as a threat, activating local effector mechanisms that may lead to sustained inflammation (Nieuwenhuizen, 2016). The transcriptomic analysis, here described, revealed that the *A. pegreffii* larvae respond to the presence of iDCs primarily through an initial transcriptional pattern, likely reflecting an early attempt to cope with human derived immune-mediated stress. Notably, *A. pegreffii* is able to regulate a complex network of metabolic pathways, strategically investing energy in activating and upregulating genes associated with energy production (i.e., cGMP-dependent protein kinase, sodium/calcium exchangers, trehalase), as well as nitrogen and amino acid metabolism (e.g., aspartate aminotransferase, histidine ammonia-lyase, amino acid transporters). This metabolic shift likely provides the necessary resources to sustain vital functions under host-induced stress (Stryiński et al., 2024). However, this response is energetically costly and may not be sustainable in the long term. The sustained metabolic demand may deplete internal energy reserves leading to the accumulation of toxic metabolic waste products, and ultimately resulting in the death of the invading *Anisakis* larva.

Interestingly, this upregulation of metabolic genes appears closely linked to the enhanced expression of genes involved in cuticle maintenance. The synthesis of its components is an energy-intensive process that requires a steady supply of amino acids and efficient protein biosynthesis mechanisms (Johnstone, 1994). The modulation of structural genes (e.g., cuticle collagen dpy-7, forming homology 2, myosin heavy chain 6) is crucial for cuticle formation (Johnstone, 2000). This serves multiple functions, including acting as a protective barrier between the nematode and its environment, maintaining its body shape (Johnstone et al., 1992), and facilitating movement through connections with muscle tissue (Francis and Waterston, 1991; Hresko et al., 1999). These processes are pivotal for nematode survival, enabling interactions with hostile environments, when trying to penetrate the gastric and intestinal submucosa (Johnstone, 1994; Page et al., 2014; Sandhu et al., 2021). Over time, the expression of genes encoding these structural proteins has likely evolved to support both developmental processes and defense mechanisms (Kim et al., 2018). For instance, Kim et al. (2018) reported that collagen-related genes were abundantly transcribed in the *Anisakis* L3, reflecting the active molting process. Similarly, Trumbić et al. (2021) observed that the genes involved in molting, such as structural constituents of the cuticle, collagen, and cuticulin, were upregulated in *A. pegreffii* L3 infecting rats, suggesting that molting-related gene expression can be influenced by host-specific factors. In this context, in the presence of iDCs, *A. pegreffii* L3 stage larvae may perceive them as potential threats, triggering a defense response characterized by the coordinated activation of metabolic, antioxidant, and structural pathways. This upregulation may represent a protective strategy aimed at shielding the nematode from host immune effectors, potentially enhancing its survival in an accidental host. However, the direct involvement of structural gene modulation —particularly collagen and cuticulin genes— remains speculative. Further functional validation is required to confirm their role in human host–parasite interactions.

Interestingly, alongside the upregulation of structural genes involved in cuticle formation, we observed the downregulation of genes associated with procollagen-proline 4-dioxygenase activity, critical for the hydroxylation of proline residues during collagen maturation (Page and Johnstone, 2007). The simultaneous upregulation of cuticle-related genes and downregulation of procollagen-proline 4-dioxygenase activity might suggest a complex regulatory mechanism in which *A. pegreffii* actively synthesizes structural precursors but limits their full post-translational maturation. However, this interpretation remains speculative, and further molecular and functional analyses are required to confirm the regulatory dynamics involved.

Moreover, the downregulation of genes involved in cytoskeletal organization (e.g., actin filament, bundle assembly) and membrane dynamics (e.g., membrane-to-membrane docking) suggests a strategic suppression of processes related to cellular motility and intracellular trafficking. This downregulation may reflect an attempt to conserve energy by limiting metabolically expensive activities that are not immediately critical for the parasite development.

Alongside this metabolic shift, genes involved in oxidative stress responses and those linked to ion homeostasis (sodium/calcium exchangers, anion exchange proteins), may play a role in modulating the host immune response (Nawaratna et al., 2018). This mechanism could contribute to mitigating the effects of reactive oxygen species (ROS) generated during the immune response, potentially enhancing parasite survival within the host. This suggests that *A. pegreffii* not only adjusts its metabolic activity, but also engages calcium-dependent pathways and antioxidant-related mechanisms to counteract host-induced oxidative stress. The modulation of antioxidant enzymes is a key strategy for parasites to defend themselves against the host immune system, specifically targeting ROS generated by immune cells such as macrophages, neutrophils and eosinophils. Indeed, our previous studies have shown that iDCs strongly increase ROS production as part of their immune defense against *A. pegreffii* (Napoletano et al., 2018). In addition, the increased transcription of genes implicated in the detoxification of ROS and bolstering antioxidant defense, the upregulation of glutathione peroxidase, thioredoxin reductase 1, peroxidase mlt-7 and superoxide dismutase suggest a metabolic pathway of *A. pegreffii* to shield itself from ROS produced by host tissue-resident cells, besides iDCs. For example, glutathione peroxidase plays a critical role in removing harmful ROS by catalyzing the reduction of hydrogen peroxide and organic hydroperoxides in *A. pegreffii*. Instead, superoxidase dismutase has been shown to be exploited in resistance to radiation (Seo et al., 2006) and xenobiotics (Stryiński et al., 2022, 2024), suggesting a versatile role of antioxidant enzymes in the parasite’s defense strategy against various environmental stressors. In the case of thioredoxin reductase, recent studies in filariasis have shown that inhibiting this enzyme induces apoptotic death of the parasite (Sen et al., 2023), highlighting its crucial role in the parasite’s survival and, consequently, in the disease pathogenesis and progression. Additionally, it is interesting to note that the thioredoxin reductase exerts an immunosuppressive role by downregulating the “inflammasome” pathways in macrophages exposed to the parasite (Joardar et al., 2021).

In addition to antioxidant enzymes, the modulation of molecular chaperones represents another critical mechanism employed by *A. pegreffii* to manage iDC-induced stress. Among these, HSP70 plays a key role in stabilizing proteins during stress (Hartl, 1996). Beyond its role in protein maintenance, HSP70 is involved in differentiation, protection against host-induced damage (including oxidative stress from free radicals), and promoting virulence (Polla, 1991; Cruz-Laufer et al., 2025). These functions allow parasites to adapt to various host niches, endure immune responses, and respond to environmental changes. In this context, HSP70 also emerges as a potential virulence factor of *A. pegreffii*. The up-regulation of the *Hsp70* gene in *A. pegreffii* co-cultured with iDCs can be understood as an adaptation to changing environments and host-parasite interactions over time. Additionally, it is noteworthy that HSP70 was detected as being carried by EVs released by L3 larvae, along with other proteins involved in metabolism and immune modulation (Palomba et al., 2023). Other specific genes were also found in EVs of *A. pegreffii* to be differentially regulated following exposure to iDCs (**Supplementary Table S4**). Beyond their role in stress responses and parasite adaptation, HSP70s are also recognized allergens in various arthropods, including mites, midges, flies, and cockroaches. Similarly, HSPs (including HSP β-1, HSP - 12.2, HSP70, HSP 75KDa, and HSP 90-α), along with superoxide dismutase and galectin, have been identified in the larval extract of the ascarid *Toxocara canis* (de Silva et al., 2018). In the *larva migrans* of *T. canis*, HSPs serve as key immunomodulatory molecules, playing essential roles in the parasite survival, even during accidental human infection (Pérez-Morales and Espinoza, 2015).

Finally, *Anis10*, *Anis11*, and *Anis12*—previously described as allergens—were detected among the differentially expressed genes (Audicana and Kennedy, 2008; Mattiucci et al., 2017). However, their specific roles remain unclear, although they are likely closely interconnected with the host’s immune response, and future investigations are essential to elucidate the precise functions of these genes and their coded proteins (Castrignanò et al., 2007).

## Conclusions

This study provides the first comprehensive transcriptomic overview of *A. pegreffii* L3 larvae during *in vitro* exposure to human iDCs, revealing for the first time a coordinated activation of antioxidant enzymes, molecular chaperones, and structural genes in response to early immune recognition. The responses triggered by the parasite likely reflect a short-term survival strategy against immune-mediated stress in a non-permissive host environment. Notably, the upregulation of detoxification enzymes and molecular chaperones, along with the modulation of cuticle-related genes, highlights the parasite’s attempt to mitigate host-induced oxidative stress and maintain structural integrity. However, the concurrent downregulation of genes necessary for collagen maturation and cellular motility suggests that while *A. pegreffii* initiates defense mechanisms, it may be unable to sustain the energetic cost of adaptation in the human host.

Overall, the molecular characterization of the *A. pegreffii* L3 stage larvae, interacting with iDCs here described, appear to be consistent with the parasite’s inability to develop. At the same time, our data show that the larva activates a transcriptional response likely aimed at counteracting the immune pressure exerted by iDCs — an early, but ultimately unsuccessful, attempt to ensure survival.

While previous work by Napoletano et al. (2018) has shown that *A. pegreffii* L3 can impair DC function by inducing apoptosis and blocking their differentiation, maturation, and migration to lymph nodes, our study focuses on the parasite’s point of view — specifically, how the larva itself responds to contact with human iDCs at the transcriptomic level. The expression patterns observed suggest that the parasite attempts to resist immune stress by activating genes involved in energy metabolism, oxidative stress response, and structural maintenance. This may allow temporary adaptation to the host environment and could contribute indirectly to shaping the local immune response. In particular, the modulation of DC activity by the parasite — as reported in our previous studies (Napoletano et al., 2018) — may influence Th1/Th2 polarization, potentially favoring a shift toward a Th2-biased environment, typical of chronic helminth infections (Sher et al., 2003).

Further studies are needed to explore whether the upregulated genes identified in this analysis influence the host immune response. For instance, specific antigens derived from these genes could be tested *in vitro* to assess their immunomodulatory potential, as already done in other parasite models, such as *Trichinella* sp*iralis* (Yu et al., 2025). In this context, human organoid systems represent a promising experimental platform to investigate the host–parasite interaction in a physiologically relevant setting. These 3D models can mimic the structure and function of the human gastrointestinal epithelium and allow co-culture with immune cells (Perez et al., 2025). They could be used to assess barrier integrity, local cytokine responses, and immune cell activation, as well as to explore whether *A. pegreffii* antigens contribute to chronic inflammation or tumor-promoting conditions, particularly in the context of prolonged or repeated exposure (Bellini et al., 2024).

## Data Availability

The datasets generated for this study can be found in the NCBI (National Center for Biotechnology Information) under the accession number PRJNA752284 and in Figshare (see **Table 3**).
