# Supplementary material for: Transcriptomic profile of the zoonotic parasite Anisakis pegreffii upon in vitro exposure to human dendritic cells
Source: Front Cell Infect Microbiol. 2025 Sep 15;15:1646537. doi: 10.3389/fcimb.2025.1646537 (PMC12477248; doi:10.3389/fcimb.2025.1646537)
Supplement: Supplementary file 1 [file DataSheet1.docx]

Supplementary Material

**S1 File.** Dendritic cells (DCs) generation

Immature DCs (iDCs) and mDCs were collected on day 5 and phenotypically characterized (Napoletano et al., 2007; Dionisi et al., 2018) to assess iDCs quality before the setting up of the co-culture with L3 larvae. DCs phenotype was performed by flow cytometry employing monoclonal antibodies (MoAbs) directly conjugated with fluorescein isothiocyanate (FITC) or phycoerythrin (PE): IgG1 or IgG2b FITC and IgG1 or IgG2b PE, as isotype controls; anti-CD14-PE, anti-CD83-PE anti CD40-PE, anti-CD86-FITC, anti-HLAII-DR (FITC) anti-CCR7-FITC, matched fluorochrome isotype controls were employed. Briefly, after washing with PBS, 105 DCs were incubated with the fluorescence-conjugated MAbs (1 hour, 4°C), washed thricely with PBS and sample analyzed with FACSCcanto II, Becton Dickinson running DIVA software. Results were evaluated as percentage of positive cells or as mean fluorescence intensity (MFI) after subtraction of isotype control values. iDCs were defined with CD14-DR+CD83-CD86+; mDCs were defined as CD14-DR++CD83+CD86++.
