# Supplementary material for: Transcriptomic profile of the zoonotic parasite Anisakis pegreffii upon in vitro exposure to human dendritic cells
Source: Front Cell Infect Microbiol. 2025 Sep 15;15:1646537. doi: 10.3389/fcimb.2025.1646537 (PMC12477248; doi:10.3389/fcimb.2025.1646537)
Supplement: Supplementary file 2 [file DataSheet2.docx]

Supplementary Material


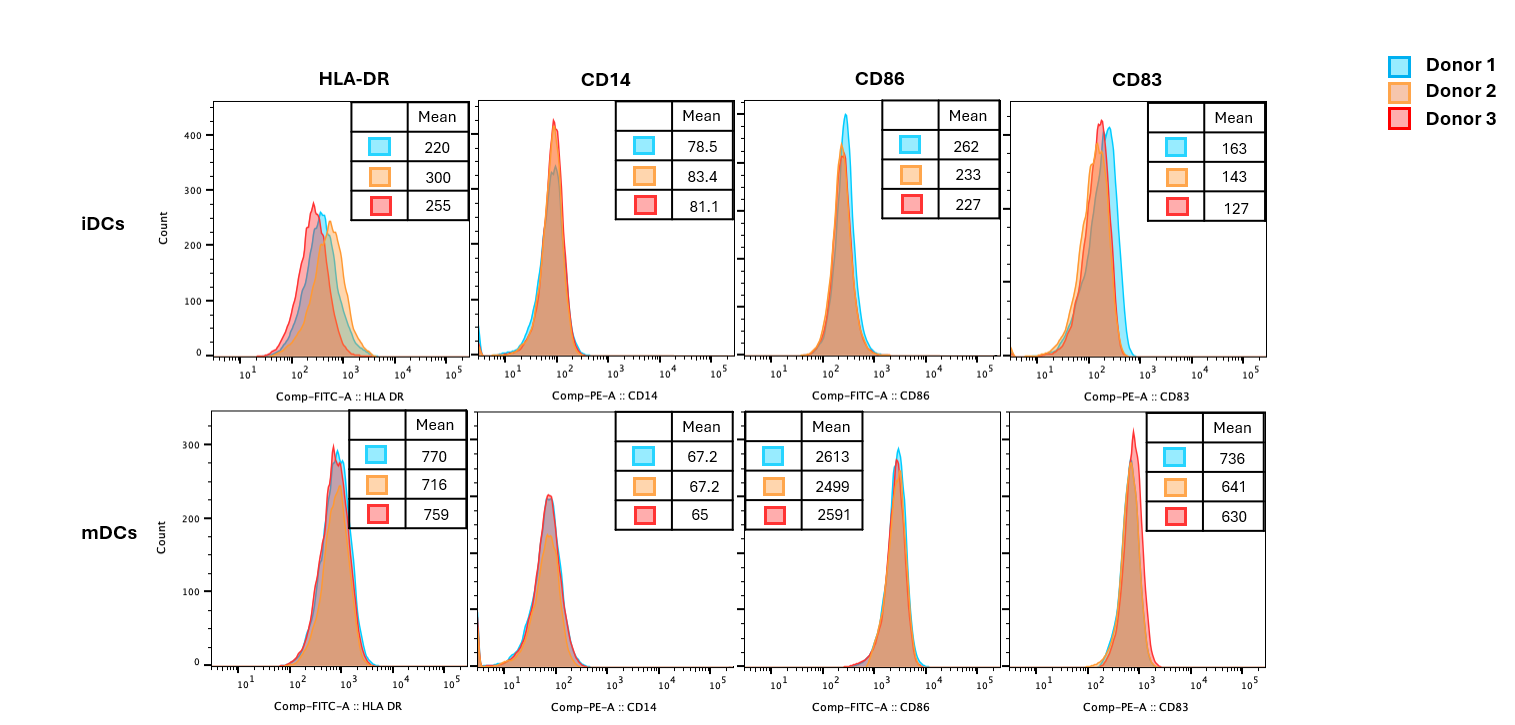


**Figure S1**. Cytofluorimetric analysis of iDCs and mDCs phenotype. DCs culture were generated from three halthy donors (Donor 1: Light blu square; Donor 2: light orange square; Donor 3: light red square). To obtain mature DCs (mDCs) maturation cytokine cocktail (PGE2, TNFa, IL1b) was added to iDCs at day 4 for 24 hours. Phenotype was performed at day 5 for iDCs and mDCs. Histograms represent the fluorescence signal associated to each marker while tables showed the mean fluorescence intensities (MFI) of each marker. HLA-DR and CD86 correspond to costimulatory molecules, that are up-regulated after maturation; CD14 is a monocyte marker that remains negative in iDCs and mDCs; CD83 is a specific marker of DC maturation and is upregulated upon maturation.


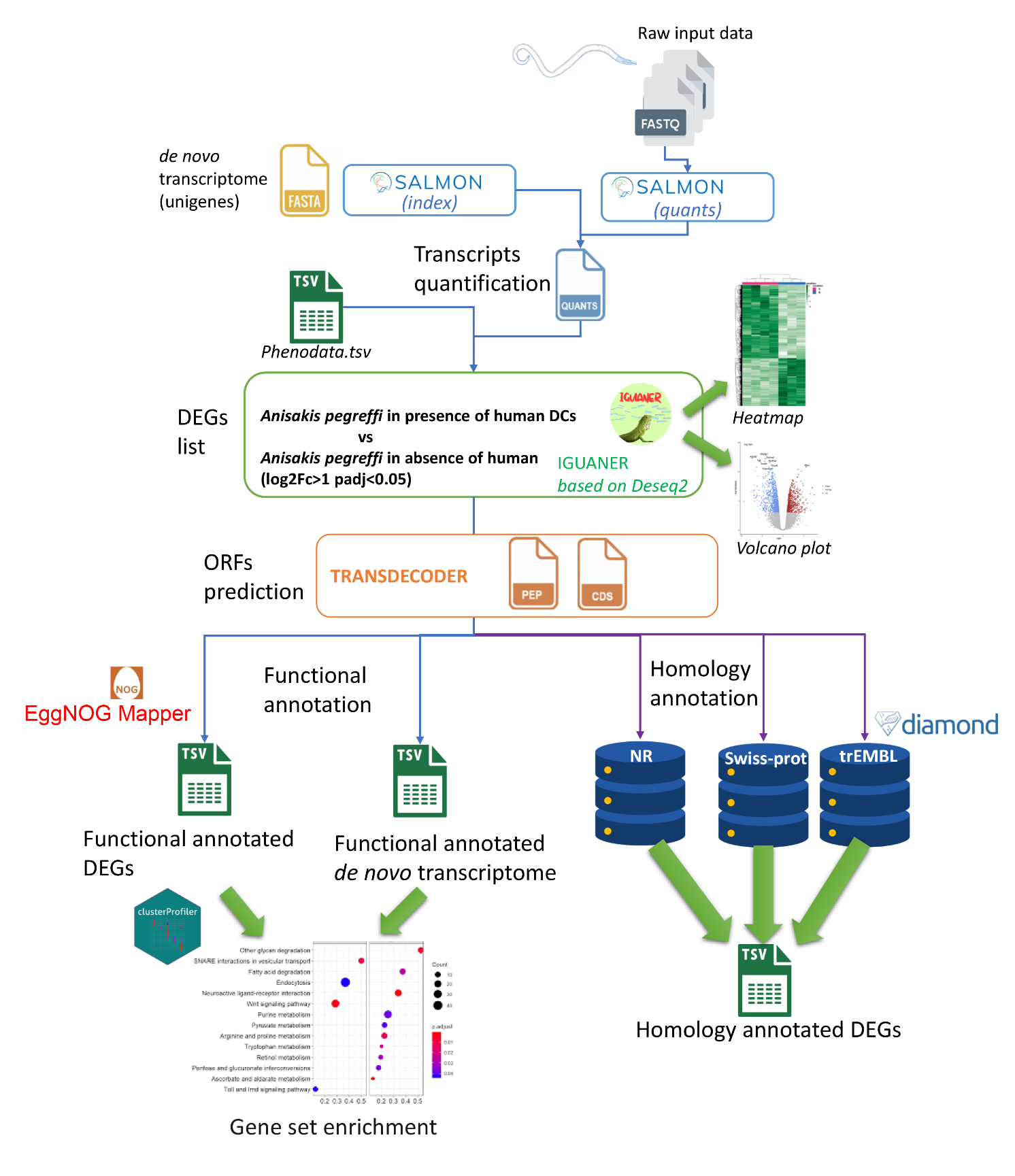


**Figure S2**. Workflow of bioinformatic pipeline.


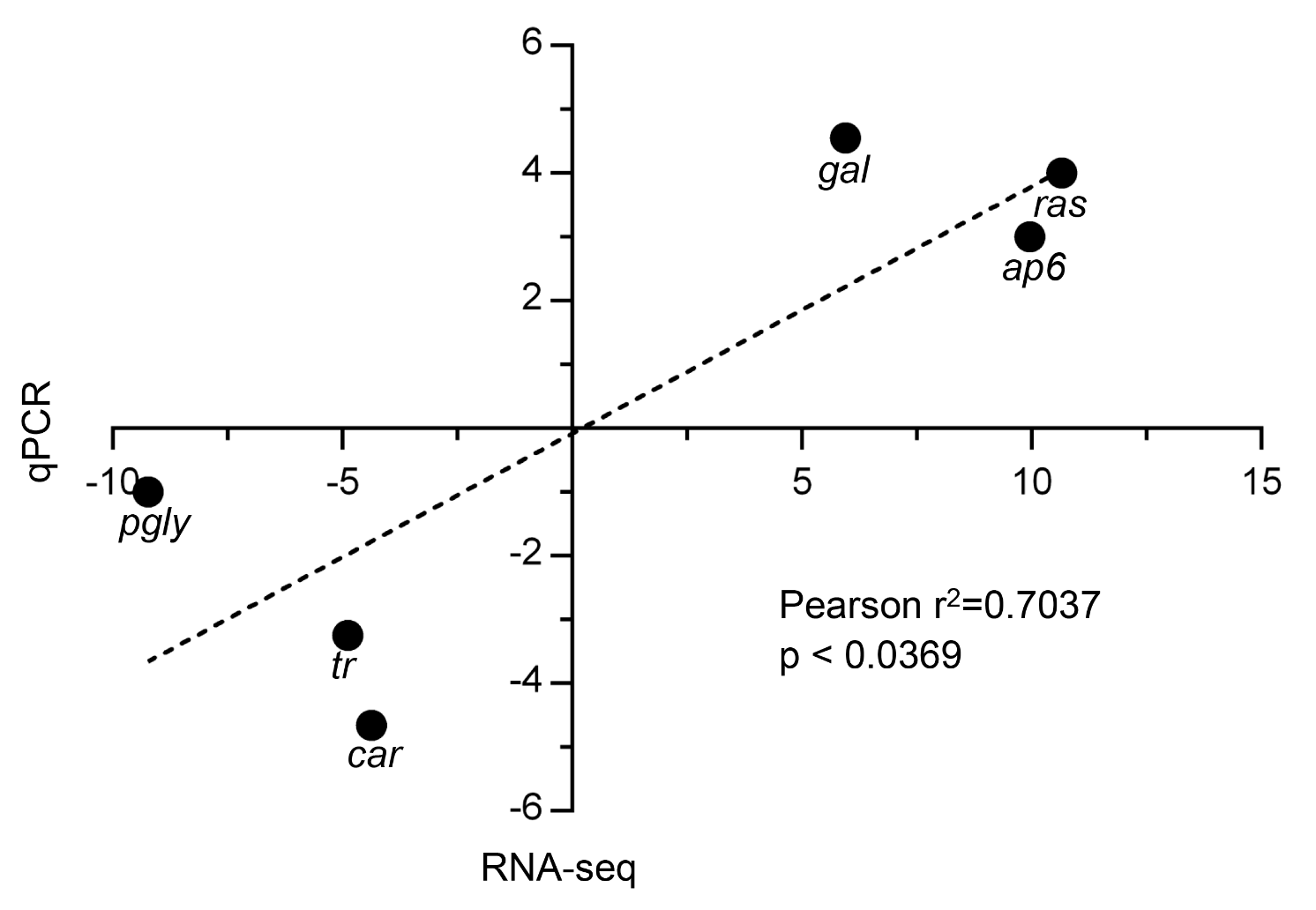


**Figure S3**. RT-qPCR validation. Correlation between the transcriptional abundance of 6 genes in *A. pegreffii* L3 (p-glycoprotein 2, tetraspanin, carboxypeptidase, ras-related protein Rab, galectin, aspartic protease 6) in the presence/absence of DCs, as revealed by RT-qPCR and RNA-seq. The level of abundance is defined as the ratio between each sample value over the group median (mean FPKM and mean ΔCt for RNA-seq and RT-qPCR data, respectively) in both RT-qPCR and RNA-seq approaches. For both techniques, statistical evaluation was performed throughout the Pearson test. EF gene was used as the internal control.
