# Supplementary material for: Transcriptomic profile of the zoonotic parasite Anisakis pegreffii upon in vitro exposure to human dendritic cells
Source: Front Cell Infect Microbiol. 2025 Sep 15;15:1646537. doi: 10.3389/fcimb.2025.1646537 (PMC12477248; doi:10.3389/fcimb.2025.1646537)
Supplement: Supplementary file 3 [file Table1.docx]

**Table S1.** Primer sequences of selected genes used in this study.

| **Gene** | **Primer name** | **Primer sequence (5’ – 3’)** | **Length (bp)** |
| --- | --- | --- | --- |
| P-glycoprotein 2 | PGLY2F | GCAGTCGAAGAGAACTGGCT | 121 |
|  | PGLY2R | TTTCTACGACGTCAACCGGG |  |
| Carboxypeptidase | CAR-F | GAACCCCATGGATGTTCCGT | 103 |
|  | CAR-R | CCGGCACCCTTGACTGTTAT |  |
| Galectin | GAL-F | TCGACCCTCACTCGGTACAT | 145 |
|  | GAL-R | ATCGCTGGGGTGTTGAAGAG |  |
| Aspartic protease 6 | AP6-F | AAGAGTTTACCAGGCCGCTC | 133 |
|  | AP6-R | TGCACTCCCGGTATCGAGTA |  |
| Tetraspanin | TR-F | GGAATCGATGGCCTTGTTGC | 113 |
|  | TR-R | ACGACAGAGGCAGCAGAAAA |  |
| Ras-related protein Rab | RAS-F | TCTTTTAATGGCGTCGCGGA | 103 |
|  | RAS-R | ACCAACTGCACTTTCACCGA |  |
