# Supplementary material for: Transcriptomic profile of the zoonotic parasite Anisakis pegreffii upon in vitro exposure to human dendritic cells
Source: Front Cell Infect Microbiol. 2025 Sep 15;15:1646537. doi: 10.3389/fcimb.2025.1646537 (PMC12477248; doi:10.3389/fcimb.2025.1646537)
Supplement: Supplementary file 5 [file Table3.docx]

| **Table S3.** Enriched Gene Ontology (GO) terms associated with up-regulated and down-regulated genes. | | | | |
| --- | --- | --- | --- | --- |
|  |  |  |  |  |
|  |  | **GO terms** | **Description** | **Adjusted p-value** |
| Up-regulated | BP | GO:0040002 | collagen and cuticulin-based cuticle development | 9.14e-06 |
|  |  | GO:0006103 | 2-oxoglutarate metabolic process | 9.14e-07 |
|  |  | GO:0009078 | pyruvate family amino acid metabolic process | 1.68e-05 |
|  |  | GO:0140448 | signaling receptor ligand precursor processing | 2.42e-06 |
|  |  | GO:0070592 | cell wall polysaccharide biosynthetic process | 2.58e-05 |
|  |  |  |  |  |
|  | CC | GO:0031225 | anchored component of membrane | 0.037 |
|  |  | GO:0099060 | integral component of postsynaptic specialization membrane | 0.001 |
|  |  | GO:0060103 | collagen and cuticulin-based cuticle extracellular matrix part | 0.001 |
|  |  | GO:0030428 | cell septum | 2.64e-06 |
|  |  | GO:0098591 | external side of apical plasma membrane | 0.000 |
|  |  |  |  |  |
|  | MF | GO:0008239 | dipeptidyl-peptidase activity | 4.69e-27 |
|  |  | GO:0004185 | serine-type carboxypeptidase activity | 0.000 |
|  |  | GO:0047635 | alanine-oxo-acid transaminase activity | 2.89e-05 |
|  |  | GO:0042329 | structural constituent of collagen and cuticulin-based cuticle | 0.000 |
|  |  | GO:0071916 | dipeptide transmembrane transporter activity | 0.000 |
|  |  |  |  |  |
| Down-regulated | BP | GO:0051017 | actin filament bundle assembly | 0.049 |
|  |  | GO:0089718 | amino acid import across plasma membrane | 0.049 |
|  |  | GO:0044557 | relaxation of smooth muscle | 0.049 |
|  |  | GO:0022614 | membrane to membrane docking | 0.048 |
|  |  |  |  |  |
|  | CC | GO:0032421 | stereocilium bundle | 0.042 |
|  |  | GO:0008230 | ecdysone receptor holocomplex | 0.000 |
|  |  | GO:0060103 | collagen and cuticulin-based cuticle extracellular matrix part | 0.032 |
|  |  | GO:0005971 | ribonucleoside-diphosphate reductase complex | 0.002 |
|  |  |  |  |  |
|  | MF | GO:0004879 | nuclear receptor activity | 0.001 |
|  |  | GO:0003854 | 3-beta-hydroxy-delta5-steroid dehydrogenase activity | 0.000 |
|  |  | GO:0016841 | ammonia-lyase activity | 1.27e-05 |
|  |  | GO:0004656 | procollagen-proline 4-dioxygenase activity | 0.015 |
|  |  | GO:0004748 | ribonucleoside-diphosphate reductase activity | 0.002 |
